# P-glycoprotein and Mrp1 collectively protect the bone marrow from vincristine-induced toxicity *in vivo*

**DOI:** 10.1038/sj.bjc.6601363

**Published:** 2003-10-28

**Authors:** O van Tellingen, T Buckle, J W Jonker, M A van der Valk, J H Beijnen

**Affiliations:** 1 Department of Clinical Chemistry, The Netherlands Cancer Institute (Antoni van Leeuwenhoek Huis), Plesmanlaan 121, 1066 CX Amsterdam, The Netherlands; 2 Division of Experimental Therapy, The Netherlands Cancer Institute (Antoni van Leeuwenhoek Huis), Plesmanlaan 121, 1066 CX Amsterdam, The Netherlands; 3 Department of Animal Pathology, The Netherlands Cancer Institute (Antoni van Leeuwenhoek Huis), Plesmanlaan 121, 1066 CX Amsterdam, The Netherlands; 4 Slotervaart Hospital, Department of Pharmacy and Pharmacology, The Netherlands Cancer Institute, Louwesweg 6, 1066 EC Amsterdam, The Netherlands

**Keywords:** stem cells, knockout mice, pharmacokinetics

## Abstract

ABC transporter proteins may protect haematopoietic progenitor cells from chemotherapy-induced toxicity. By using an *in vitro* colony-forming assay, we found that bone marrow of *Mdr1ab*, *Mrp1*, *Mdr1ab/Mrp1* knockout (KO) mice was two-, five- to 10- and 25-fold, respectively, more sensitive to vincristine than wild-type mice bone marrow. To study the impact of ABC transporters on *in vivo* bone marrow sensitivity without the added complication of altered pharmacokinetics, we created chimeras of wild-type mice transplanted with bone marrow from wild-type, *Mrp1*, *Mdr1ab* or *Mdr1ab/Mrp1* KO donor mice. Following a single bolus injection of vincristine, the chimeras transplanted with wild-type or *Mdr1ab* KO marrow cells showed no reductions in WBC. A significant reduction was observed in *Mrp1* KO chimeras, but the most pronounced effect was observed in mice receiving bone marrow from *Mdr1ab/Mrp1* KO mice. A pharmacokinetic analysis in wild-type and KO mice showed that the absence of P-gp reduced the body clearance of vincristine, but that no further reduction occurred when Mrp1 was also absent. However, the tissue accumulation of vincristine in tissues of these *Mdr1ab/Mrp1* KO mice was further increased. This study demonstrates that the presence of multiple drug transporters protects the bone marrow, and probably other tissues as well, against chemotherapeutic insults.

ABC transporters such as P-glycoprotein (P-gp) provide efficient protection against xenobiotics. As demonstrated by using knockout (KO) mouse models, P-gp and Breast cancer resistance protein (Bcrp1) limit the uptake from the gastrointestinal tract of substances, such as drugs (Sparreboom *et al*, 1997; Jonker *et al*, 2000) or potentially toxic dietary substances (Jonker *et al*, 2002). Similarly, P-gp in the blood–brain barrier limits the entry of substances into the brain (Schinkel *et al*, 1994, 1996). Multidrug resistance-associated protein (Mrp1) KO mice were hypersensitive to etoposide (Lorico *et al*, 1997; Wijnholds *et al*, 1997). Mrp1 protects the oropharyngeal mucosal layer and the testicular tubules (Wijnholds *et al*, 1998) and plays a role in the blood–cerebrospinal fluid barrier (Rao *et al*, 1999; Wijnholds *et al*, 2000).

Myelotoxicity is a common complication when treating cancer patients with chemotherapeutic drugs. Already in 1991, Chaudhary and Roninson (1991) showed that P-gp was present in haematopoietic progenitor cells, suggesting that this transporter might also be involved in the protection of bone marrow stem cells. The role of drug transporters in bone marrow-derived cells under *in vitro* conditions was further addressed in several studies *in vitro*. *Mdr1ab* P-gp contributed to the extrusion of the fluorophore rhodamine 123 from haematopoietic progenitor cells (Schinkel *et al*, 1997). Moreover, it was recently shown that Bcrp1 is the transport protein responsible for the extrusion of the marker dye Hoechst 33342 (Zhou *et al*, 2001; Kim *et al*, 2002; Scharenberg *et al*, 2002). Flow cytometry cell sorting of the fraction of Hoechst 33342 dull cells resulted in a highly enriched fraction of primitive haematopoietic progenitor cells. Subsequent RT–PCR analyses of RNA from these so-called side-population (SP) cells revealed the presence of *Mrp1*, *Mrp3* and *Mrp4* but not *Mrp2* (Zhou *et al*, 2001).

The role of these drug transporters *in vivo* is currently under investigation. Bcrp1 appears to protect the bone marrow from mitoxantrone-induced toxicity (Zhou *et al*, 2002). Johnson *et al* (2001) showed that mice with compound disruptions of the *Mdr1a*, *Mdr1b* and the *Mrp1* genes (*Mdr1ab/Mrp1* triple KO mice) were about 128-fold more sensitive for i.p. vincristine. Their results suggested that the bone marrow might be involved in the toxicity profile, but it remained unclear whether the toxicity was directly due to the absence of these drug transporters in the haematopoietic progenitor cells or whether it was also, or even merely, a consequence of a reduced drug clearance in these *Mdr1ab/Mrp1* triple KO mice. The pharmacokinetics of vincristine has not been documented, but it is well established that the absence of P-gp reduces the clearance of substrate drugs, such as vinblastine (van Asperen *et al*, 1996).

In the present study, we have investigated the role of P-gp and Mrp1 in the protection of the bone marrow *in vitro* and *in vivo*. We have used a bone marrow transplantation model to avoid complications in data interpretation due to differences in drug clearance. Wild-type mice receiving whole-body irradiation at a dose that was lethal to the bone marrow were transplanted with bone marrow from donor mice deficient in *Mdr1ab* and/or *Mrp1* genes. Animals transplanted with wild-type bone marrow were used as control group. After their full recovery, the mice were exposed to the anticancer drug vincristine and the bone marrow toxicity was determined by serial analyses of haematology parameters. In parallel experiments, the pharmacokinetics and toxicology of vincristine in the wild-type and *Mdr1ab/Mrp1* triple KO mice were established using a selective high-performance liquid chromatographic (HPLC) assay.

## MATERIALS AND METHODS

### Animals

Male wild-type, *Mrp1* knockout (Wijnholds *et al*, 1997), *Mdr1ab* double knockout (*Mdr1ab* DKO; animals with compound deletion of *Mdr1a* and *Mdr1b* genes) (Schinkel *et al*, 1997) and *Mdr1ab/Mrp1* triple knockout (*Mdr1ab/Mrp1* TKO) were used. The latter were obtained by crossbreeding of *Mrp1* KO and *Mdr1ab* DKO mice. All mouse strains were backcrossed for at least seven generations to obtain a more than 99% homogeneous FVB background. The animals were given food (Hope Farms BV, Woerden, The Netherlands) and acidified water *ad libitum*. They were handled according to the institutional guidelines, which are based on Dutch law and conform the standards required by the UKCCCR guidelines (Workman *et al*, 1998). The animal experiment committee of the Institute approved the experiments described in this paper.

### Determination of the maximum tolerated dose (MTD)

Vincristine (Pharmachemie, Haarlem, The Netherlands; 1 mg ml^−1^) was diluted in saline and administered i.v. to wild-type and *Mdr1ab/Mrp1* TKO mice, aged 10–14 weeks, at dose levels ranging between 0.125 and 4 mg kg^−1^. Animals were monitored daily and killed when they lost more than 20% of their initial body weight. The MTD was defined as one dose step below the dose where more than one animal in that group had to be killed. Necropsies were performed in wild-type and *Mdr1ab/Mrp1* TKO mice receiving vincristine at or near the MTD and killed 2 days later.

### Pharmacokinetics

The pharmacokinetic behaviour of vincristine in wild-type, *Mdr1ab* KO and *Mdr1ab/Mrp1* TKO mice, 10–14 weeks of age, was established at a dose of 1 mg kg^−1^. Animals were killed at 5, 15, 30 min, 1, 2 and 4 h after drug administration for collection of plasma. At 1 and 4 h, a range of tissues was also collected. Vincristine levels in plasma and tissues were analysed by HPLC developed by us previously (Boven *et al*, 1999). Plasma AUC_0–4 h_ (area under the curve) values were calculated by the linear trapezoidal rule using standard equations (van Asperen *et al*, 1996) and the clearance was calculated as Dose/AUC_0–4 h_. Statistical tests were performed with SPSS v11.0 (SPSS Inc., Chicago, IL, USA).

### *In vitro* bone marrow toxicity

The toxicity of vincristine in haematopoietic progenitor cells was tested by an *in vitro* colony forming unit (CFU) assay. Mouse bone marrow progenitor cells were obtained from the femurs of FVB mice flushed with Dulbecco's phosphate-buffered saline solution. After centrifugation (10 min, 200 **g**, ambient temperature), the cell pellet was resuspended in Iscoves medium (MDM) (StemCell Technologies Inc., Vancouver, BC, Canada) with 2% (v v^−1^) fetal calf serum (FCS). Nucleated bone marrow cells were seeded in Methocult GF M3534 at a density of 2.5 × 10^5^ cells ml^−1^ and vincristine was added at concentrations ranging from 2.5 to 100 ng ml^−1^. Aliquots of 1 ml were plated in duplicate in uncoated six-wells culture plates (Greiner, Alphen a/d Rijn, The Netherlands) and incubated for 5–6 days at 37^o^C in 5% CO_2_ in humidified air. CFUs were scored by phase-contrast light microscopy. We have used the concentration that reduces the number of colonies by more than 90% relative to untreated controls (IC_90_) instead of IC_50_ because the decreasing size of the colonies at higher drug concentrations makes accurate scoring of their numbers difficult. Hardly any colonies were present at the IC_90_ making this parameter easier to determine.

### *In vivo* bone marrow toxicity

Wild-type mice of about 5–6 weeks of age received whole-body irradiation of 6.8 Gy (HF320 Radiobiology Constant Potential X-ray Unit, Pantak, East Haven, CT, USA). The next morning each mouse was i.v. injected with 1.5 to 3 × 10^6^ nucleated bone marrow cells from donor mice of wild-type, *mrp1* KO, *Mdr1ab* DKO or *Mdr1ab/Mrp1* TKO genotype. After a recovery period of 6 weeks, the mice received an i.v. bolus injection of 2 mg kg^−1^ of vincristine via the tail vein. To minimise the effects of mild dehydration occurring at this dose level of vincristine, mice were supported by daily i.p. administrations of 1 ml of saline: dextrose 5% (1 : 1; v/v) for 3 days. At days 0 (before vincristine), 2, 4, 7 and 12, peripheral blood was sampled from the tail and haematologic parameters (WBC and Haemoglobin (Hb)) were determined on a Cell Dyn 1200 analyzer (Abbott Laboratories, Santa Clara, CA, USA). Experiments were performed on six different occasions. At some of these occasions, the mice receiving bone marrow of TKO mice were also challenged with vincristine at lower dose levels (e.g. 0.5 or 1 mg kg^−1^). Bone marrow was obtained at the end of the experiments from a number of randomly selected mice and used to determine the *in vitro* sensitivity of the grafted bone marrow using the *in vitro* bone marrow toxicity test.

### PCR analyses

As a random test, the genotype of the engrafted bone marrow was verified by PCR analyses in about 50% of all animals. DNA was prepared from whole-blood samples of recipient animals using the DNeasy kit (Qiagen GmbH, Hilden, Germany), according to the manufacturer's protocol. Disruption of genes in the KO mice strains was achieved by replacement of relevant genomic fragments by a selection gene, for example, hygromycin in case of *Mdr1a* (4). As a result, PCR verification of the *Mdr1a* KO genotype was verified by the presence and absence of bands for hygromycin and *Mdr1a*, respectively.

## RESULTS

We first examined the relative sensitivities of bone marrow derived from wild type, *Mrp1* KO, *Mdr1ab* DKO and *Mdr1ab/Mrp1* TKO mice using an *in vitro* CFU assay. Cell kill occurred in a dose-dependent manner. The bone marrow of wild-type animals was most resistant to vincristine (Table 1

**Table 1:** Toxicity of bone marrow progenitor cells (*in vitro*)

**Conc. (ng ml^−1^)**	**Wild type**	***Mrp1* KO**	***Mdr1ab* DKO**	***Mdr1ab/Mrp1* TKO**
100	−	−	−	−
50	+	−	−	−
25	+	−	+	−
10	+	±	+	−
5	ND	+	ND	−
2	ND	+	ND	±
1	ND	+	ND	+

Bone marrow cells harvested from wild-type, *Mrp1* KO, *Mdr1ab* DKO and *Mdr1ab/Mrp1* (TKO) mice were incubated continuously with various concentrations of vincristine. Colonies were scored by day 5. Wells were scored with − when the number of colonies was reduced by more than 90% relative to control wells without the drug (IC_90_). ± indicates a borderline value, whereas clearly less than 90% reduction is marked by a + sign. ND means not determined.

). *Mdr1ab* DKO bone marrow cells were about two-fold more sensitive, whereas *Mrp1* KO cells were about five to 10-fold more sensitive. The absence of both drug transporters in cells from the *Mdr1ab/Mrp1* TKO mice resulted in an about 25-fold higher susceptibility towards vincristine. These experiments were repeated several times and although the absolute IC_90_ values varied with different batches of culture medium, the relative differences in IC_90_ between the bone marrow of the various genotypes were consistent throughout these experiments.

We next investigated the MTD of vincristine in our mouse strains (Table 2

**Table 2:** Toxicity of vincristine in mice

				**Decrease in body weight**		
	**Dose (mg kg^−1^)**	**Number of animals**	**Toxic deaths**	**Median (%)**	**Range**	**Day of nadir**
*Mdr1ab/Mrp1* TKO	0.125	9	0	1.9	0–7.9	2
	0.25	13	0	3.0	0–7.4	3
	0.5	9	0	10.3	4.1–19.1	4
	1	4	2	20.5	14.1–27.2	3–4
						
Wild ype	1	5	0	4.6	3.6–7.6	2
	2	5	1	12.1	10.9–20.6	2
	4	5	5	23.1	21.0–25.1	4

Animals received vincristine by i.v. injection. Body weight was determined daily. Animals experiencing more than 20% body weight loss were killed and counted as toxic deaths. The size of, especially, the highest dose groups was kept as low as possible in order to minimise the number of animals experiencing major discomfort.

). Based on our previous experience, we selected a few dose levels in wild-type animals (1, 2 and 4 mg kg^−1^). The MTD in wild-type mice was 2 mg kg^−1^, where the mice experienced mild but clear signs of toxicity, including pilo-erection, and a median weight loss of about 12% of the initial value achieved around day 2, followed by weight gain thereafter. Only one wild-type mouse receiving 2 mg kg^−1^ experienced body weight loss in excess of 20%; however, this nadir was reached at 7 days after drug administration at a time that surviving animals were already regaining weight. It was not clear whether this event was due to vincristine toxicity or to other causes. A dose level of 4 mg kg^−1^ was clearly above the MTD, since five out of five animals experienced more than 20% body weight loss.

The MTD in *Mdr1ab/Mrp1* TKO mice was clearly lower at 0.5 mg kg^−1^, but similar macroscopic signs of toxicity were observed. At a dose of 1 mg kg^−1^, two out of four animals had to be killed (at days 4 and 5) because their body weight dropped by more than 20%, whereas the two others survived. These results indicate that there is a moderate increase in vincristine toxicity in animals lacking both these drug-transporting proteins.

Toxic effects of vincristine in wild-type and *Mdr1ab/Mrp1* TKO mice were assessed by full necropsy of the animals 2 days after they received vincristine at their respective MTD. In *Mdr1ab/Mrp1* TKO mice receiving 0.5 mg kg^−1^ toxicities manifested by arrested mitoses were found in the intestines, skin, bone marrow, the adrenal medulla and in the bases of the incisors. No evidence for central nervous toxicity or liver toxicity was observed. In wild-type mice receiving a dose of 2 mg kg^−1^, the incisors were affected and a moderate depletion of the bone marrow was seen, but no signs of toxicity were found in any of the other tissues, except for some mild vacuolisation of spinal ganglion neurons.

**Figure 1:**
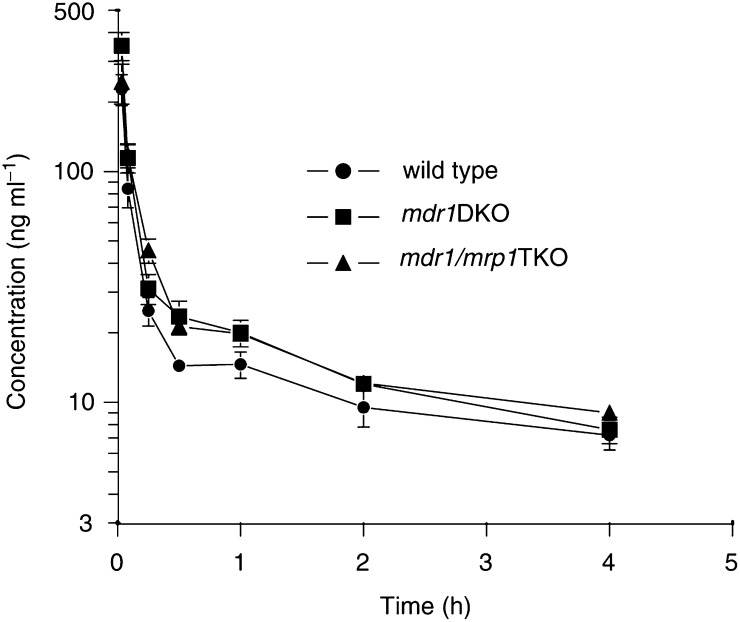
Plasma concentration–time profiles of vincristine. Mice received 1 mg kg^−1^ of vincristine by i.v. injection. Each time point represents at least four animals. The AUC values were calculated by the linear trapezoidal rule and were 61.8±3.8, 83.1±4.2 and 82.4±3.4 ng ml^−1^ h for wild type, *Mdr1ab* DKO and *Mdr1ab/Mrp1* TKO, respectively. The latter two were significantly higher (*P*<0.01) than the values of wild-type mice, but not significantly different from each other.

**Figure 2:**
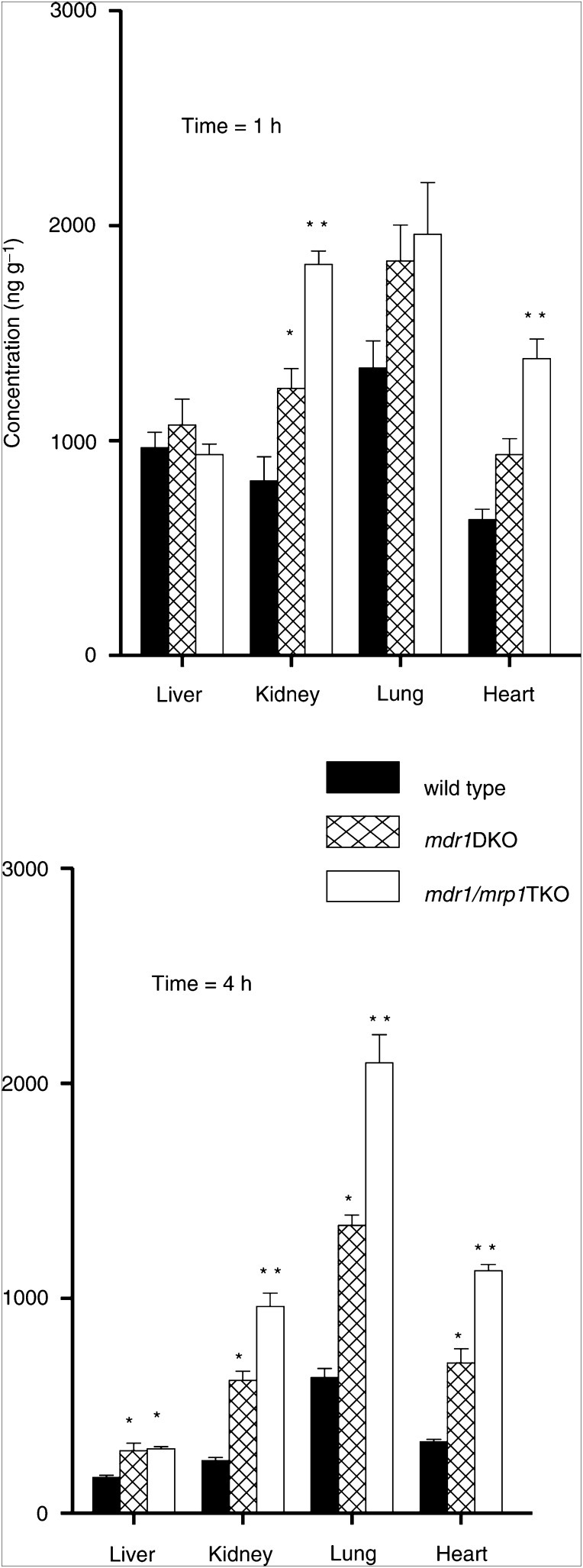
Tissue accumulation of vincristine. Mice receiving 1 mg kg^−1^ were killed at 1 and 4 h after drug administration. Drug levels were determined by HPLC. Each bar represents at least four animals and the error bar depicts the s.e. Statistical analyses were performed by ANOVA using Bonferroni *post hoc* test for multiple comparisons. ^*^ *P*<0.05 relative to wild-type; ^**^ *P*<0.05 relative to *Mdr1ab* DKO, otherwise not significant.

We further investigated the pharmacokinetics of vincristine given at a dose of 1 mg kg^−1^ to wild-type, *Mdr1ab* DKO and *Mdr1ab/Mrp1* TKO mice by sampling of blood and tissues for up to 4 h after drug administration. Although this dose level was above the MTD in *Mdr1ab/Mrp1* TKO mice, it was selected because toxic effects are relatively mild during the first 4 h and because of the detection limit of the assay (5 ng ml^−1^ for a 200 *μ*l plasma sample). In line with the toxicity results there was only a moderate reduction in the plasma clearance of vincristine (Figure 1

), and this appears to be due mainly to the absence of P-gp, since the plasma concentration–time curves of *Mdr1ab* DKO and *Mdr1ab/Mrp1* TKO mice are overlapping. The plasma clearance in wild-type mice was 16.2±1.0 *μ*g ml^−1^ h and was significantly (*P*<0.01) reduced to 12.0±0.6 and 12.1±0.5 *μ*g ml^−1^ h in *Mdr1ab* DKO and *Mdr1ab/Mrp1* TKO mice, respectively. Similar as in plasma, the vincristine levels observed in tissues of *Mdr1ab* DKO were significantly higher than in wild-type mice (Figure 2

). Interestingly, however, the levels of vincristine in several tissues of *Mdr1ab/Mrp1* TKO were again significantly higher than those in *Mdr1ab* DKO, despite equivalent plasma levels, suggesting that Mrp1 offers additional protection against vincristine accumulation in these tissues. The vincristine levels in the brain were below the limit of detection in all animals.

To avoid the complicating effects of these transporters on the pharmacokinetics of vincristine, we investigated the consequences of the absence of the various transporters on the bone marrow *in vivo* using animals with transplanted bone marrows of different genotype. By using this approach we ensured that the only difference in these mice was in the genotype of their bone marrow. Wild-type mice, which tolerate the highest dose of vincristine, received whole-body irradiation at a dose of 6.8 Gy. All mice that were not supplemented with donor bone marrow died within 10 days after irradiation when the WBC and the Hb concentration had dropped to extremely low values. With a few exceptions, the mice that did receive bone marrow of donor mice remained in good condition. These results show that irradiation induced bone marrow ablation had occurred and was the primary cause of death. Wild-type animals received donor bone marrow of either wild-type, *Mrp1* KO, *Mdr1ab* DKO or *Mdr1ab/Mrp1* TKO mice, and were allowed to recover for an additional 6 weeks before receiving one cycle of vincristine chemotherapy by i.v. administration. Most animals were treated with the MTD of vincristine for wild-type mice (2 mg kg^−1^).

**Figure 3:**
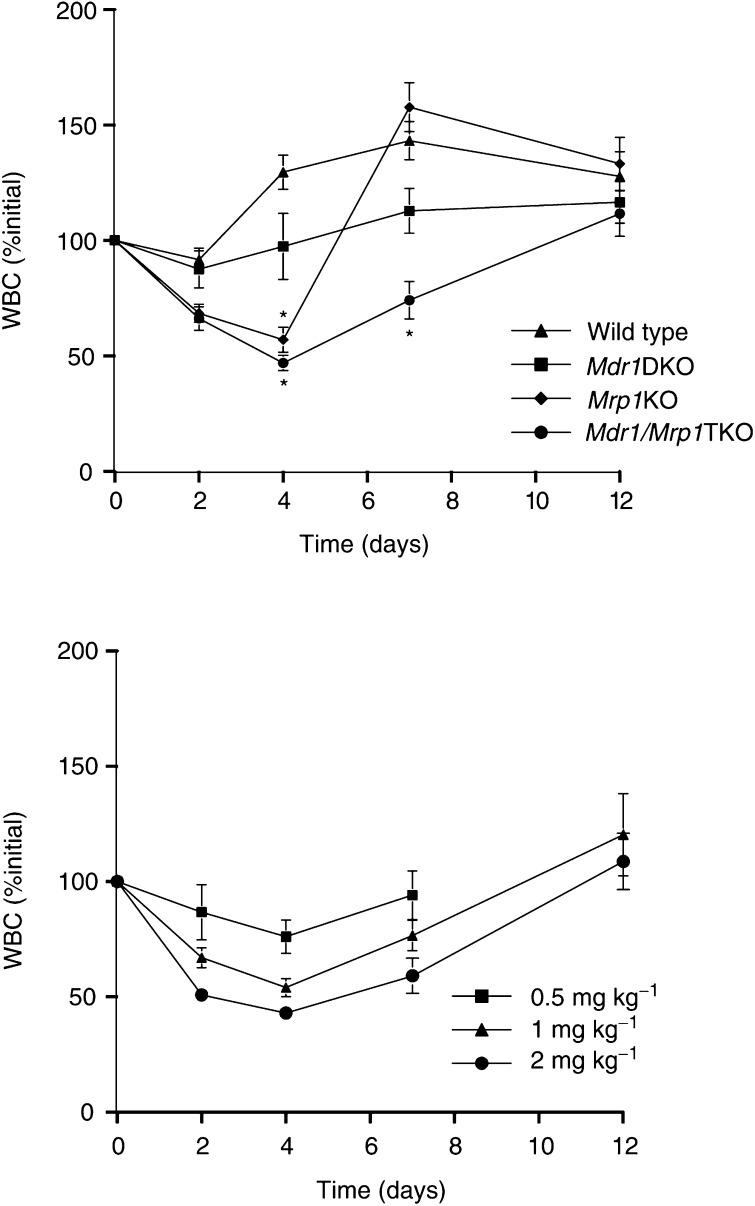
*In vivo* bone marrow toxicity induced by vincristine: effects on WBC. Lethally irradiated wild-type mice, which had received donor marrow from wild-type (*n*=38), *Mrp1* KO (*n*=33), *Mdr1ab* DKO (*N*=18) and *Mdr1ab/Mrp1* TKO (*n*=38) mice, were challenged with 2 mg kg^−1^ of vincristine administered by i.v. injection (upper panel). The course of the WBC in blood is depicted as percentage of the initial value at day 0 (arbitrarily set at 100%). ^*^ *P*<0.05 relative to wild type, otherwise not significant. The lower panel depicts the results in *Mdr1ab/Mrp1* TKO donor marrow grafted mice receiving 0.5 mg (*n*=15), 1 mg (*n*=20) or 2 mg (*n*=21) of vincristine per kilogram and show the dose-dependent response on WBC.

Both in wild type and in *Mdr1ab* DKO chimeras, there was no reduction in WBC (Figure 3

). On the contrary, the levels increased after day 2, which may be due to chemotherapy-induced recruitment of cells from the bone marrow or due to the procedure of blood sampling from the tail vein; a procedure that may cause a mild inflammatory response. The WBC counts in animals receiving *Mrp1* KO bone marrow had dropped significantly at day 4, but showed a steep rebound by day 7. The most pronounced effect on WBC was observed in mice receiving *Mdr1ab/Mrp1* TKO donor marrow. The nadir in these mice occurred at day 4 followed by a gradual recovery to baseline values within about 12 days. The nadir in WBC in *Mdr1ab/Mrp1* chimeras was clearly dose dependent (Figure 3: right panel).

**Figure 4:**
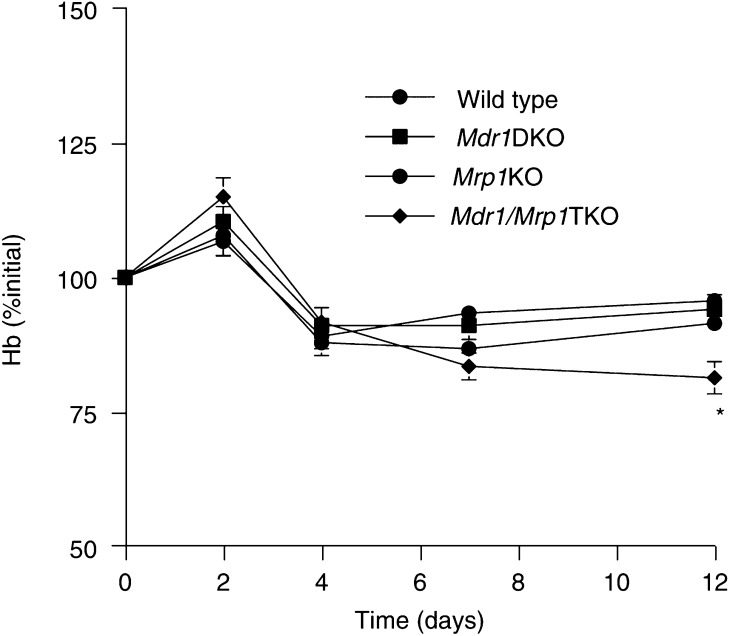
*In vivo* bone marrow toxicity induced by vincristine: effects on Hb levels. Lethally irradiated wild-type mice received donor marrow from wild-type, *Mrp1* KO, *Mdr1ab* DKO and *Mdr1ab/Mrp1* (TKO) mice were challenged by i.v. administration of 2 mg of vincristine per kilogram (left panel). The course of the haemoglobin concentration is depicted as percentage of the initial value at day 0 (arbitrarily set at 100%). ^*^ *P*<0.05 relative to wild type; otherwise not significant.

The Hb concentrations in wild-type and *Mdr1ab* KO chimeras increased slightly by day 2, which may be due to the mild dehydration as a consequence of the toxicity occurring at this dose of vincristine (Figure 4

). In *Mdr1ab/Mrp1* TKO chimeras receiving a lower dose (0.5 or 1 mg kg^−1^) of vincristine, this effect was not observed (not shown). By day 12, the Hb level in *Mdr1ab/Mrp1* TKO chimeras was significantly lower than observed in the other cohorts (*P*<0.001). Overall, however, the effect on Hb was less severe than the effect on the WBC counts, which is probably due to the longer half-life of mature red blood cells compared to peripheral WBC.

We performed a random check whether the repopulation of the bone marrow by donor mice was complete or only partial by harvesting of bone marrow of animals at the end of the study, which was and in the *in vitro* CFU assay. In all cases, bone marrow derived from *Mdr1ab/Mrp1* chimeras showed the same high sensitivity to vincristine as bone marrow obtained from *Mdr1ab/Mrp1* TKO mice used as control. In addition, PCR analyses of whole-blood samples from the mice at the end of the experiment were used to verify the genotype of the donor bone marrow. Although the results for the hygromycin gene (selection marker for the *Mdr1a* KO allele) were consistent in all cases, we observed a weak signal for *Mdr1*a in mice that received bone marrow of *Mdr1ab* DKO or *Mdr1ab/Mrp1* TKO donor mice. This is probably due to the high sensitivity of the PCR assay, which detects small quantities of host genomic DNA in the blood. It was estimated that up to 5% of circulating nucleated cells were of wild-type origin.

## DISCUSSION

This study using an *in vivo* model shows that the ABC-transporters P-gp and Mrp1 protect haematopoietic progenitor cells in the bone marrow from vincristine toxicity. The most significant effects in WBC and Hb values were observed in mice grafted with bone marrow from *Mdr1ab/Mrp1* TKO mice. *Mrp1* chimeras showed intermediate signs of haematotoxicity, whereas no toxicity was observed in animals that received bone marrow from *Mdr1ab* DKO or wild-type donor mice. Thus, in the case of vincristine, the two transporters appear to function in concert in bone marrow progenitor cells and the alternate transporter can partly or completely compensate the loss of function of the other. Overall, the lethality in *Mdr1ab/Mrp1* TKO chimeras after challenging with vincristine was a few percent and not higher than in animals bearing *Mdr1ab* DKO, *Mrp1* KO or wild-type bone marrow. This shows that the increased haematotoxicity after single high-dose vincristine treatment was not a dose-limiting event *per se*.

A pharmacokinetic analysis of vincristine showed that, relative to wild-type mice, the plasma clearance of vincristine was reduced in *Mdr1ab* DKO mice but not further reduced in *Mdr1ab/Mrp1* TKO mice. This result is in line with the location of P-gp in apical membranes of excretory organs (liver, intestines, kidneys) (Thiebaut *et al*, 1987), where it is involved in detoxification by extrusion of substrates from the body, whereas Mrp1 is mainly located in basolateral membranes and has relatively little direct effect on drug excretion from the body (Borst *et al*, 1999; Hipfner *et al*, 1999). However, this study shows that the accumulation of vincristine in tissues from *Mdr1ab/Mrp1* TKO mice was clearly enhanced relative to *Mdr1ab* DKO mice showing that Mrp1 offers protection to tissues. A previous study in *Mrp1* KO mice did not find any effects on tissue distribution of etoposide (Wijnholds *et al*, 1997). This discrepancy may be (partly) due to the presence of P-gp in these mice, which may be such a dominant factor that it conceals the effects of a loss of Mrp1. However, it is also possible that the analytical methodology (determination of total radioactivity after administration of radiolabelled drug) was not suited to find these differences, because it cannot discern unchanged substrate drug from (radiolabelled) metabolites and/or degradation products. We have used a selective HPLC to determine unchanged vincristine levels. The higher drug levels in tissues correlates with previous reports of local organ toxicities when *Mrp1* KO mice were challenged with chemotherapeutics (Wijnholds *et al*, 1998). It thus seems likely that P-gp located at the apical side and Mrp1 at the basolateral side of epithelia cooperate in limiting the accumulation of compounds that are substrates of both transporters, such as vincristine, whereas they are less efficient for compounds that are substrate of only one of them. Moreover, their concerted activity has also been shown in cells expressing these transporters in a nonpolarised fashion. By using immortalised fibroblast cell lines, it was found that both *Mdr1ab* and *Mrp1* are implicated in innate resistance to cytotoxic substrates (Allen *et al*, 2000; Lin *et al*, 2002), but the strongest effects were found in cells lacking both transporters. Our findings of a markedly enhanced toxicity by vincristine in the *in vitro* bone marrow toxicity assays are in line with these results.

The dose-limiting toxicity of vincristine in *Mdr1ab/Mrp1* TKO mice appears to be related to the effects on the gastrointestinal tract. We found that the *Mdr1ab/Mrp1* TKO mice were only about four-fold more sensitive to vincristine than wild-type mice, which is much less than the 128-fold reported previously (Johnson *et al*, 2001). It is unlikely that the different routes of administration in the two studies (i.p. *vs* i.v.) would explain this discrepancy. In our experience, the clearance of hydrophobic drugs from the peritoneal cavity of mice occurs rapidly. Moreover, both wild-type and *Mdr1ab/Mrp1* TKO mice received vincristine by the same route, and the results for wild-type mice were consistent between the two studies. The difference may be explained by differences in drug elimination between animals in the two studies. In addition to elimination by excretion, metabolism is also a very important route for many drugs including vincristine. It has previously been shown that *Mdr1a* KO mice maintained in our institute have a higher expression of cytochrome P450 isoenzymes than mice of similar genotype kept in the United States (Schuetz *et al*, 2000). Consequently, clearance of vincristine might occur more rapidly in our populations of *Mdr1ab* DKO and *Mdr1ab/Mrp1* TKO mice, rendering them less susceptible to vincristine.

To eliminate the effects of reductions in body clearance of vincristine on bone marrow toxicity, we have used a bone marrow transplantation model yielding chimeras that were of identical wild-type genotype, except for their bone marrow progenitor cells. The course of the WBC counts in the *Mdr1ab/Mrp1* TKO chimeras closely resembled the pattern that is usually observed in patients experiencing chemotherapy-induced myelotoxicity. The nadir and return to baseline levels occurred somewhat more abruptly, but this is most likely due to the fact that most physiological processes in mice proceed at a higher velocity than in humans. Interestingly, the nadir was about similar in *Mrp1* KO chimeras, but there was a very strong rebound in WBC counts by day 7. This difference between *Mrp1* KO and *Mdr1ab/Mrp1* TKO chimeras may be due to the fact that the relative expression of ABC transporters appears to vary along the lineage from uncommitted stem cells to mature blood cells. In mice, CD34^−^ sorted SP cells appear to represent a more primitive subpopulation of progenitor cells than CD34^+^ cells (Osawa *et al*, 1996) and these cells contain relatively high levels of *Bcrp1* and *Mdr1* mRNA, whereas *Mrp1* appears to be higher in the murine CD34^+^ subpopulation of cells (Zhou *et al*, 2001). The absence of Mrp1 will make this population of CD34^+^ progenitor cells more vulnerable to vincristine. A single bolus of a high dose of vincristine given to *Mrp1* KO chimeras may significantly reduce the numbers of these precursor cells, thus eliminating part of the maturing blood cells from the pipeline. The decline in WBC will trigger signalling to induce a compensatory wave of haematopoiesis and because the population of primitive CD34^−^ precursor cells is probably not so much affected due to protection by P-gp, this can still occur effectively. In *Mdr1ab/Mrp1* TKO chimeras, however, this more primitive population of CD34^−^ cells is no longer protected by P-gp and may therefore be more vulnerable to vincristine, thus causing further delay in WBC recovery.

In this study, we have shown that a single dose of vincristine resulted in a clear but nonlethal bone marrow toxicity, which was Mrp1 and P-gp dependent. Repeated dosing or continuous infusion of this G2–M cell cycle specific drug might have resulted in greater bone marrow toxicity. More pronounced cytotoxic effects on these primitive precursor cells have been shown in a recent study, where irradiated mice that were transplanted with mixtures of wild-type and *Bcrp1* KO donor bone marrow were challenged with mitoxantrone (Zhou *et al*, 2002). After five daily repeated injections of 2 mg of mitoxantrone per kilogram, the relative contribution of *Bcrp1* null cells in the peripheral blood myeloid and lymphoid compartment declined to very low values and remained low during the many weeks afterwards. In this case with repeated dosing of mitoxantrone, it appears that the majority of primitive *Bcrp1* null stem cells have been eradicated and have been replaced by wild-type cells. Given that mitoxantrone is also a substrate of P-gp, we expect that the effect of this drug may be even greater in bone marrow cells with a compound deletion of the *Mdr1ab* and *Bcrp1* alleles.

In conclusion, although the physiological function of P-gp, Mrp1 and other ABC-transporters in haematopoietic progenitor stem cells is still conjectural, it is clear that they protect the cells against chemotherapy-induced injury. Inhibition of their function, for example, as part of drug regimens aimed to sensitise drug-resistant tumour cells, may thus result in enhanced myelotoxicity. However, the fact that multiple transporters with (partly) overlapping substrate specificities are present appears to be a safety mechanism that may render this a relatively infrequent complication.
